# THE ROLE OF RIBOSOMAL COMPOSITION IN SELECTIVE TRANSLATION OF LONGEVITY GENES UNDER DIETARY RESTRICTION

**DOI:** 10.1093/geroni/igz038.368

**Published:** 2019-11-08

**Authors:** Jarod A Rollins

**Affiliations:** MDI Biological laboratory, Bar Harbor, Maine, United States

## Abstract

Dietary restriction influences longevity through changes in gene expression on the level of transcription and translation. Our lab is investigating the changes occurring under dietary restriction in C. elegans on the level of individual ribosomal proteins (RPs) and changes in RP phosphorylation and how they shape gene expression and longevity. To look at the impact of changes in RP expression we used RNAi knockdowns starting at adulthood and assayed lifespan and health span. Two RPs knockdowns that increased longevity, rpl-7A and rpl-22, were then subjected to transcriptome and translatome analysis using polysome profiling and mRNA-seq. Both knockdowns resulted in similar transcriptomic changes with an up-regulation of genes related to translational fidelity and mitochondrion organization. In contrast, the translatome analysis showed minimal changes upon loss of rpl-7A and rpl-22 suggesting that they are not responsible for increase in selective translation previously observed under DR. To look at impact of changes in phosphorylation of rps-6 on DR, we generated mutant lines incapable of being phosphorylated by TOR signaling. These mutants were assayed for changes in lifespan, health span and selective translation. While phenotypically similar to controls under well fed conditions, the mutants were longer lived under DR. Furthermore, preliminary results indicated that the selective translation of the anti-longevity microchondrial electron transport genes cytochrome c oxidase and ubiquinol-cytochrome c reductase binding protein was repressed when rps-6 is not phosphorylated. In summary, our preliminary results that ribosomal protein phosphorylation but not protein composition is responsible for guiding selective translation pf longevity genes under DR.

